# DDeep3M+: adaptive enhancement powered weakly supervised learning for neuron segmentation

**DOI:** 10.1117/1.NPh.10.3.035003

**Published:** 2023-06-23

**Authors:** Rong Xiao, Lei Zhu, Jiangshan Liao, Xinglong Wu, Hui Gong, Jin Huang, Ping Li, Bin Sheng, Shangbin Chen

**Affiliations:** a Huazhong University of Science and Technology, Britton Chance Center for Biomedical Photonics, Wuhan National Laboratory for Optoelectronics, Wuhan, China; b The Hong Kong University of Science and Technology, Department of Electronic and Computer Engineering, Hong Kong, Hong Kong, China; c Wuhan Institute of Technology, School of Computer Science & Engineering, Wuhan, China; d Wuhan Textile University, School of Mathematics and Computer Science, Wuhan, China; e The Hong Kong Polytechnic University, Department of Computing, Kowloon, Hong Kong, China; f Shanghai Jiao Tong University, Department of Computer Science and Engineering, Shanghai, China

**Keywords:** convolutional neural network, weakly supervised deep learning, image segmentation, neuron segmentation, Hessian matrix

## Abstract

**Significance:**

Robust segmentations of neurons greatly improve neuronal population reconstruction, which could support further study of neuron morphology for brain research.

**Aim:**

Precise segmentation of 3D neuron structures from optical microscopy (OM) images is crucial to probe neural circuits and brain functions. However, the high noise and low contrast of images make neuron segmentation challenging. Convolutional neural networks (CNNs) can provide feasible solutions for the task but they require large manual labels for training. Labor-intensive labeling is highly expensive and heavily limits the algorithm generalization.

**Approach:**

We devise a weakly supervised learning framework Docker-based deep network plus (DDeep3M+) for neuron segmentation without any manual labeling. A Hessian analysis based adaptive enhancement filter is employed to generate pseudo-labels for segmenting neuron images. The automated segmentation labels are input for training a DDeep3M to extract neuronal features. We mine more undetected weak neurites from the probability map based on neuronal structures, thereby modifying the pseudo-labels. We iteratively refine the pseudo-labels and retrain the DDeep3M model with the pseudo-labels to obtain a final segmentation result.

**Results:**

The proposed method achieves promising results with the F1 score of 0.973, which is close to that of the CNN model with manual labels and superior to several segmentation algorithms.

**Conclusions:**

We propose an accurate weakly supervised neuron segmentation method. The high precision results achieved on 3D OM datasets demonstrate the superior generalization of our DDeep3M+.

## 1 Introduction

Neurons are the basic units of the structure and function of the nervous system.^1^ And the neuronal morphology reflects the function of the brain. A complete segmentation of neuronal structure from optical microscopy (OM) images is essential to reveal the connections of brain circuits and investigate synaptic integration, neuron morphology, neuron connectivity, and brain mechanisms.^2^ Analyzing the morphology and connection of neurons enables us to have a deeper understanding of the operating mechanism of brains and facilitates diagnosis of brain disease.^3^ For example, congenital nystagmus affects starburst amacrine cells,^4^ and amyotrophic lateral sclerosis affects upper and lower motor neurons.^5^ Alzheimer’s disease (AD) is a neurodegenerative disease associated with synaptic loss and neuronal degeneration.^6^ A precise segmentation of 3D neuron structures is vital to probe impaired brain functions of AD animal models and to determine early treatment strategies.^7^ We might gain a better understanding of such disorders if we could find specific neuronal morphology in disease models.^8^

Through the accurate signals of neuron morphology, we can systematically classify brain cells. In recent years, labeling techniques^9^^,^^10^ and optical imaging methods^11^^,^^12^ have made a series of breakthroughs, which can generate terabyte (TB) level neuronal morphological data at whole brain scale.^13^^,^^14^ Micro-optical sectioning tomography (MOST)^15^ and its follow-up study fluorescence micro-optical sectioning tomography (fMOST)^16^ have realized the whole brain imaging of specific neural structures at submicron resolution, including tens of thousands of complete neuronal morphologies. Therefore, it is highly difficult to extract and analyze neuron morphology in neuron tracing caused by the uneven distribution of fluorescence,^17^ differences of imaging system,^18^ complex morphology of neurons,^19^ and low signal-to-noise ratio (SNR).^20^ Currently, many efforts have been devoted to develop automatic or semi-automatic neuron tracing algorithms. However, most of the neuron tracing methods are not applicable in challenging datasets where the 3D neurites are contaminated by strong background noises or containing weak light signals. These methods degrade in performance of neuron segmentation from more complex image blocks. Therefore, an efficient segmentation method is urgently needed to reduce the impact of noises and enhance the weak neurite signals, which should improve the result of neuron tracing.

In recent years, deep learning has achieved remarkable success in processing natural images. Convolutional neural networks (CNNs) have been employed to segment tubular structures due to its capability of feature learning and its nonlinear relationship capturing between inputs and outputs. Deep learning techniques have been adopted in neuron segmentation to improve the segmentation quality. A deep learning toolbox, DeepNeuron, was designed to trace neurons with manually reconstructed neurons as training samples.^21^ The general supervised deep learning algorithms require a large number of manual labels for reaching an efficient and accurate neuron segmentation, but the segmentation labeling is pretty expensive and labor-consuming. Moreover, extensive *a priori* knowledge of neuroscience is also required for manual labels.

**Fig. 1:**
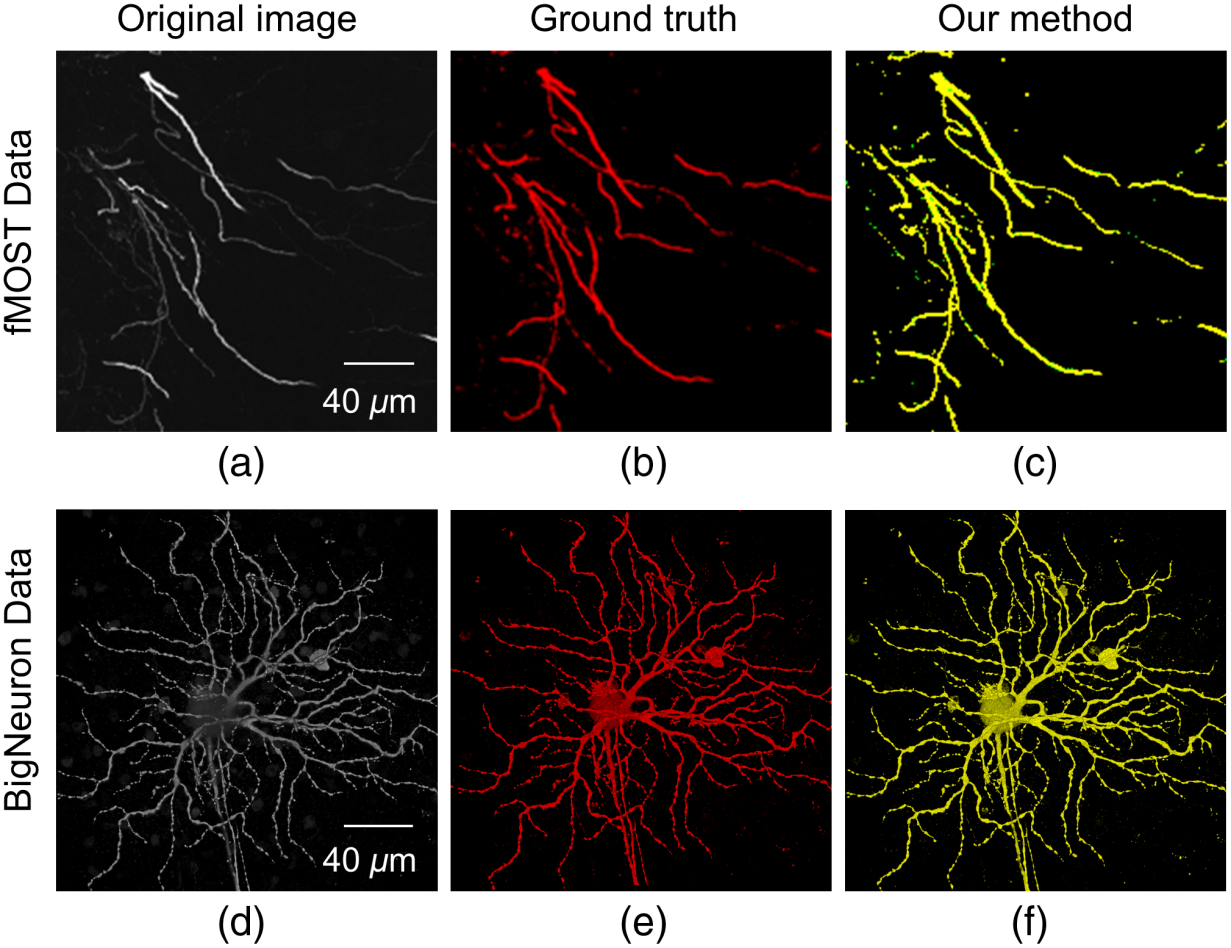
Typical segmentation result of two diverse OM image datasets, including the maximum intensity projection of fMOST^16^ and BigNeuron^22^ dataset. Red pixels indicate the ground truth. Yellow pixels represent the overlapping regions between the prediction and ground truth.

To solve the above problems, we develop a weakly supervised learning framework [Docker-based deep network (DDeep3M+)] for neuron segmentation from 3D OM images without any manual labeling. It can extract accurate neuron signals robustly from noisy backgrounds. We take advantage of both conventional methods and deep learning techniques as follows. An adaptive enhancement filter is first employed to produce pseudo-labels for training a deep segmentation neural network (DDeep3M). The proposed DDeep3M+ method improves prediction of DDeep3M by iteratively optimizing the training labels and retraining model via region growing and image fusion based on neurite structure information to mine undetected weak neurites from the probability map predicted by DDeep3M. The experimental results on the fMOST^16^ datasets and public BigNeuron^22^ datasets have demonstrated the high precision and generalization achieved by the proposed neuron segmentation method; see Fig. 1.

Our main contributions are summarized as follows:

1. We propose a precise, automatic, and general method for neuron segmentation from noisy and low-contrast OM images. And a weakly supervised learning framework based on DDeep3M network is presented to improve 3D neuron segmentation without manual labels.
2. An adaptive enhancement filter is presented in the first stage to generate pseudo-labels by enhancing weak neuronal structures based on Hessian matrix in a fibrous structure. We take advantage of adaptive enhancement filter and deep segmentation network to make them mutually complement to each other.
3. Neuron segmentation results on 3D OM datasets demonstrate the high precision and generalization by the proposed method in automatic neuron segmentation. Moreover, our DDeep3M+ outperforms state-of-the-art weakly supervised neuron segmentation algorithms on fMOST and BigNeuron datasets.

## 2 Related Work

Morphology of neurons is essential for the organization and function of the brain. Segmentation or tracing of 3D neuron structures from OM images are the main approaches to characterize and analyze neuronal morphology. There are 2,32,613 digitally reconstructed neurons contributed by 800 laboratories worldwide in NeuroMorpho^19^ dataset, which took more than 2.5 million hours of manual work in the past decades. In computational neuroscience, there are high requirements for automatic and accurate neuron segmentation methods.

With the development of optical imaging and molecular labeling technology, we can obtain large-scale sub-micron resolution neuron images through optical imaging of mammalian brain. These advances promote the generation of various optical images for different applications, and pose new challenges in neuron segmentation. The limit is the significant differences in image quality and attributes between different datasets due to a variety of factors, such as intensity range, image size, neuron structure, etc.^22^ Another challenge is that microscopy images usually have high background noise. It has difficulty in separating weak neuron voxels from an inhomogeneous background.^23^ It is tough to distinguish the neurites with low intensity and non-uniformity from the noise background, especially for the large-scale neuron images with discontinued segments of neurites and ultra-low SNR. The variability between different datasets and the low SNR of optical images increase the difficulty of generalization and improvement of neuron segmentation algorithms. Many semi-automatic or automatic methods have been proposed for neuron segmentation or neuron tracing.^24^ In these algorithms, various computational methods addressing global and local image features have been used to realize neuron segmentation and tracing. These include but are not limited to region growing, tubular model, full path pruning, graph theory method, and support vector machine based on self-learning.^25^^,^^26^ These algorithms usually show good performance on OM images with clear structure. However, the above methods are designed for specific datasets or specific problems, and their performance on different types of datasets may degrade, so complex parameter tuning is needed. In addition, most algorithms perform poorly in tracing neurites from low SNR images, and are unable to recognize weak neurite signals or prone to over-segmentation due to background noise.

Deep network^27^ has been widely used in many fields, including computer vision, natural language processing, speech recognition, and so on, and has achieved excellent results in these tasks.^28^ Since the full convolution network (FCN),^29^ various deep networks have been proposed to improve the accuracy of image segmentation, including U-Net,^30^ 3D deeply supervised network (DSN),^31^ and VoxResNet.^32^ The excellent performance of deep network encourages researchers to apply it to neuron segmentation.^33^ In Ref. 34, a deep network is designed to improve the performance of existing tracing algorithms. The main purpose of the deep network is to denoise the image containing a single neuron or multiple sparse neurons. DeepNeuron,^21^ a deep learning toolbox, is designed to trace neurons with human-reconstructed neurons as training samples. But they have poor performance in the case of weak neurites. Yang et al.^35^ employed a two-stage 3D neuron segmentation approach to segment neuron images from the BigNeuron dataset, via learning deep features by an FCN model and enhancing weak neuronal structures based on Hessian Eigenvalues in a fibrous structure. Structure-guided segmentation network (SGSNet),^36^ a two-branch network for 3D neuron segmentation, contains a shared encoding path but utilizes two decoding paths to enhance weak neuronal structures and remove background noises. There are 3D CNNs specifically designed for neuron segmentation and tracing that show better performance on images with high noise.^37^^,^^38^ However, these algorithms require a lot of labor, time-consuming, and expensive manual annotation to segment neurons. Users need to annotate enough new samples for neuron datasets from different brain regions to achieve reliable prediction. The need for manual annotation has greatly limited the promotion of deep learning-based methods for various optical neuron images.

Here, we propose a weakly supervised learning method for deep CNN without manual labeling. Integrating the advantages of traditional image enhancement and deep learning, we can extract neuron signals accurately and robustly from noisy background, and realize neuron segmentation in OM image.

## 3 Materials and Methods

**Fig. 2:**
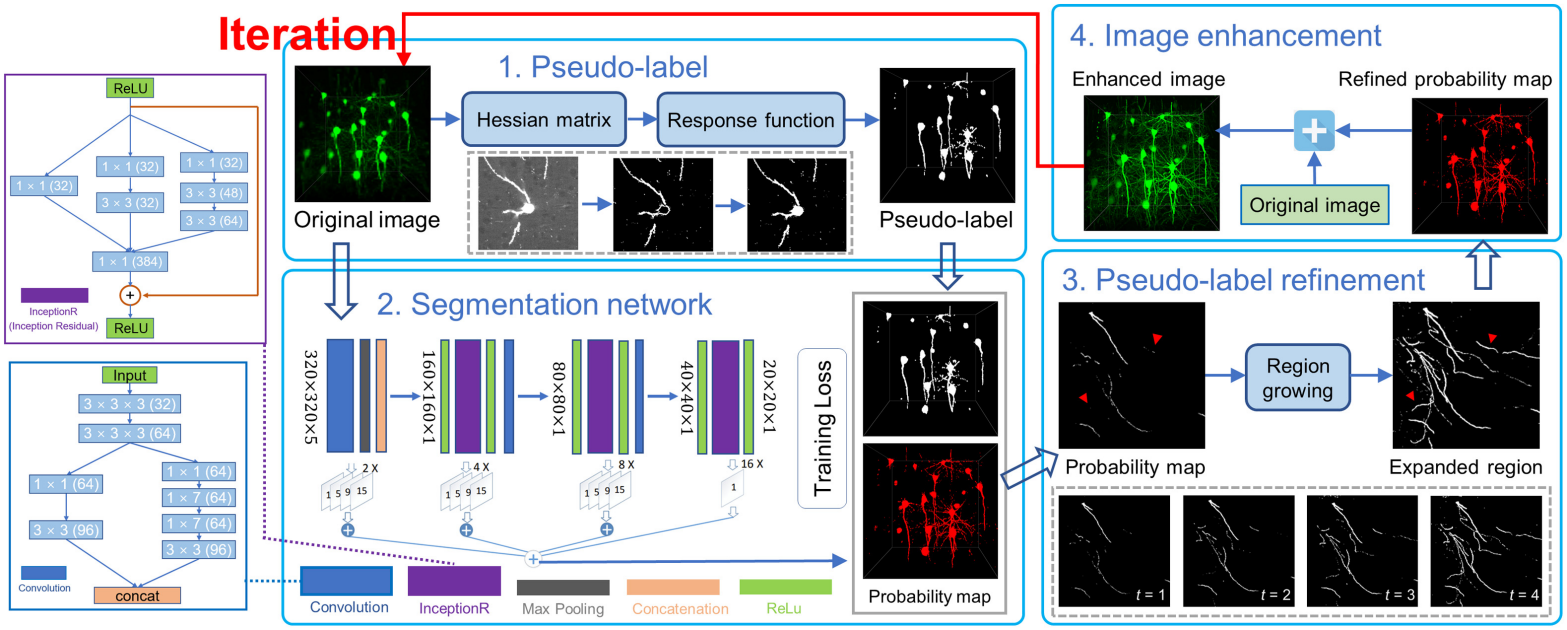
Schematic illustration of the proposed weakly supervised learning framework (i.e., our DDeep3M+) for automatic neuron segmentation. Our method consists of four steps: (1) using adaptive enhancement filter to segment the neural signal from the OM image and generate pseudo labels; (2) training and predicting of DDeep3M network with raw images and their pseudo-labels as training sets; (3) refining pseudo labels using region growing; (4) combining the prediction probability map with the original image. Iterative steps 1 to 4 update the pseudo label of training samples to improve segmentation.

To segment neuron population from low SNR optical microscopic images, our neuron segmentation framework consists of four key modules: pseudo-labels module, DDeep3M segmentation network, region growing module, and image enhancement module, which are illustrated in Fig. 2. The automatic neuron segmentation framework includes four steps: (1) initial neurite segmentation is automatically obtained by an adaptive enhancement filter and threshold segmentation algorithm from optical images as their pseudo-labels, (2) training of the DDeep3M segmentation network using input images and their pseudo-labels as training sets, and prediction of the neurite as probability map using DDeep3M, (3) refinement of pseudo-labels from probability map based on region growing, (4) combining the original image intensities with the refined probability map. Steps (1) to (4) are iterated to update the probability map. The segmentation network is optimized, and the completeness and accuracy of neuron segmentation are improved. By fusing the prediction probability map of neuron voxel with the original intensity to enhance the original image, we can preserve the global structure from probability map and local signal from original image at the same time. We gradually improve the performance of neuron segmentation by combining the traditional image enhancement method and the DDeep3M network without any manual annotation. Finally, we complete the automatic segmentation of large-scale neuronal population from noisy and low-contrast OM images through whole framework.

### 3.1 Pseudo-Labels

There are some problems in the 3D optical images of neurons, such as weak signal, strong noise, and uneven signal distribution. All these problems seriously restrict neuron segmentation, so we enhance the original image to remove the noise in the first place. Usually, a neuron consists of a soma and a great number of fibers. The fibers are formed from an axon and dendrites. Seeing a neuron in mathematical graphical structure, we can understand that a cell body is a dot-like structure and a neurite is a fiber-like or line-like structure.^39^ We propose an adaptive enhancement for the 3D image of neurons based on distance transform (DT) and Hessian matrix. The purpose of enhancement filters for neuron is to get clearer and higher contrast images, and then the neuron can be segmented after being enhanced.

The Hessian matrix is a mixed second derivate matrix, which simply indicates that the gray intensity trends of continuous voxels in a 3D image. A 3D raw OM image volume was presented as I (x,y,z). Hessian matrix was defined as the second order derivatives of the image intensity

$$H=(\nabla G⊗I)((\nabla G⊗I{)}^{T})=[\begin{array}{ccc} I_{xx} & I_{xy} & I_{xz}\\ I_{yx} & I_{yy} & I_{yz}\\ I_{zx} & I_{zy} & I_{zz} \end{array}],$$ (1)

where $G={\left(2\pi s^{2}\right)}^{-3/2}\;\exp(-(x^{2}+y^{2}+z^{2})/(2s^{2}))$ is a Gaussian kernel. ∇G denotes the first order Gaussian derivatives. ⊗ is convolution. $I_{ij}$ denotes the mixed second derivative along dimensions i and j.

First, the window size of the Hessian matrix is determined by DT: DT can measure the thickness of different neuron fibers effectively, and it can be used to calculate the Hessian matrix. Second, the method constructs a structural response function to enhance the neuron fibers: when the image signal is bright and the background is dark, the Hessian matrix eigenvalue $\lambda_{1}$ is approximately equal to 0, $\lambda_{2}$ and $\lambda_{3}$ are <0 and their amplitudes are close to each other. The method takes advantage of this characteristic to enhance the neuron fibers. Third, the method enhances the soma with different strategy: the signal value of the soma is high and the radius is large. Accordingly, this method sets a higher threshold for DT to localize the soma for hole filling. Through the above three steps, the enhancement of neuron with different structures is finally achieved. Through a Hessian analysis-based neuron enhancement and neurite threshold segmentation, the initial neurite segmentation is automatically obtained, and then they are regarded as initial training labels (pseudo-labels).

We devise a way to get an adaptive window size, which can guarantee that the Hessian matrix has a proper window size in our image of uneven intensity. First, we distinguished the foreground regions and background regions with a determined value derived from *a priori* knowledge, the image was transformed to be binary. Then we used the Euclidean DT to describe the degree of the thickness. Here, the Euclidean DT between M and N is $D(M,N)={\left(\overset{k}{\underset{i=1}{\sum }}{\left|m_{i}-n_{i}\right|}^{p}\right)}^{\left(1/p\right)}$. Here M and N are k-tuples (k=3 in this study), $m_{i}$ and $n_{i}$ are the i’th coordinates of M and N, p=2 in Euclidean distances. The purpose of computing the DT is to determine the window size of the Hessian matrix. As a second-order partial derivative matrix, the Hessian matrix needs to determine how many points to calculate when calculating between voxels. The size of the Hessian matrix window is closely related to the thickness of the nerve fibers. It has a great influence on the subsequent nerve fiber enhancement. First, the center point of a cell body is set as X. After the Euclidean DT, the DT value of the cell center point is set to DT(X). Next, the window size of the Hessian matrix is calculated by the obtained DT value.

Then the window radius is determined by the following formula: $R=\log_{2}\;\mathrm{DT}(X)$. The intensity of neurites in the middle is higher than that at the edge. DT image reveals the structure information of the graph, to a certain extent. We made use of this useful information and defined an optimal window radius R for each voxel. The DT value for each location (x,y,z) is defined as D (x,y,z), and all the DT values for each voxel in the image build a DT matrix, which has the same size as the original image. Then we normalize DT(X) between 1 and 256. We defined the normalized result for each voxel as DN (x,y,z). We make a logarithmic operation for DN, the result of it comes to be the window radius. Indeed, in this way, we basically used a window with radius R between 1 and 8. The window size, that is, window diameter DI is defined as twice the radius and plus one. The above formulas are as follows: $R=\log_{2}\mathrm{DN}(x,y,z)$, DI=2R+1. It can provide a proper size for each voxel for the uneven gray-scale characteristics of different locations in neurites to calculate the Hessian matrix.

Since the Hessian matrix is symmetric, there exist three eigenvalues $\lambda_{1}$, $\lambda_{2}$, and $\lambda_{3}$. For convenience, they are sorted based on the absolute value: $\left|\lambda_{1}|\leq |\lambda_{2}|\leq |\lambda_{3}\right|$. In previous studies, such as Frangi’s^40^ method in 1998 when the signal is bright and the background is dark, the Eigenvalues of an ideal 3D dot-like, line-like, and plate-like area satisfy the following conditions: line-like: $\lambda_{1}\approx 0$; $\lambda_{2}\approx \lambda_{3}<0$; dot-like: $\lambda_{1}\approx \lambda_{2}\approx \lambda_{3}<0$; plate-like: $\lambda_{1}\approx \lambda_{2}\approx 0$; $\lambda_{3}<0$. Initially, taking account of the neurite enhancement, Frangi’s filter proposed three derived parameters based on the above-mentioned Eigenvalues: $p_{1}=|\lambda_{2}|/|\lambda_{3}|$, $p_{2}=\frac{\left|\lambda_{1}\right|}{\sqrt{\left|\lambda_{2}\lambda_{3}\right|}}$, and $p_{3}=\sqrt{\lambda_{1}^{2}+\lambda_{2}^{2}+\lambda_{3}^{2}}$. The response function was defined as follows:

$$(X)=\{\begin{array}{ll} 0, & \text{if}\;\lambda_{2}>0\;\text{or}\;\lambda_{3}>0\\ (1-e^{-ap_{1}^{2}})e^{-bp_{2}^{2}}(1-e^{-\frac{p_{3}^{2}}{c}}), & \text{else} \end{array}$$ (2)

where a, b, and c are thresholds that control the sensitivity of the line filter to the measures $p_{1}$, $p_{2}$, and $p_{3}$. Through the test of multiple groups of data, we found the optimal values of parameters: a=5:55, b=2, and $c=2\times 10^{6}$. When a voxel in the image is a point in a plate-like structure, $\left(1-e^{-ap_{1}^{2}}\right)$ approaches 0. The calculation result of O(X) is 0 without response. When a voxel is a point in a dot-like structure, $e^{-bp_{2}^{2}}$ approaches 0. The result of O(X) is 0 without response. When a voxel is background signal, $\left(1-e^{-p_{3}^{2}/c}\right)$ approaches 0. The result is with no response. Therefore, nerve fibers can be specifically enhanced by the Eq. (2).

The above work is for line-like structures enhancement, the soma of a neuron will be turned into a circular ring or an irregular shape with a hole,^41^ as shown in Step 1 of Fig. 2. To solve the problem and make our algorithm complete and useful, we must design a method to detect the holes. In the original fluorescent neuron image, the intensity of the soma and middle part of the fiber is always higher than the other pixels. We take advantage of this feature and combine the characteristic of DT, then design the following approach to detect the holes in soma and thicker fiber. First, we choose a relatively higher determined value $I_{2}$, which is bigger than $I_{1}$, with *a priori* knowledge. Then, we recalculate DT matrix with the higher threshold $I_{2}$, defined as $D_{2}$, the result will show the location of the holes in soma and fiber. We can use the location to fill the hole in the enhanced image by replacing it with high intensity at the same location. If $D_{2}(X)>T$, O(X)=255; otherwise, it remains O(X). Where X is the voxel, $D_{2}(X)$ is the local DT value for X with higher threshold, T is the threshold to determine whether a voxel is within the soma area or not. And O(X) is the enhanced result before soma detection. If the voxel is assumed as a soma voxel, we replace the intensity value. Otherwise, the previous enhanced result will be retained. The O(X) is the enhanced result of original image, and then we obtain the initial training labels (pseudo-labels) through threshold segmentation after enhancement by our Hessian analysis based adaptive enhancement filter.

### 3.2 Segmentation Network—DDeep3M

Docker is an open source lightweight virtualization technology. DDeep3M^42^ integrates the CDeep3M^43^ network by Docker, and it can run on the local computer by downloading a separate Docker image file containing all necessary libraries, software and framework. DDeep3M greatly reduces the threshold for biomedical researchers to accept deep learning technology. It achieves excellent segmentation performance on multiple datasets and performs better than most related models, especially in the neuroscience image segmentation tasks.

The architecture of DDeep3M has some similarities to the U-Net architecture,^30^ which consists of a bottom-up path and a resolution recovery path. We aggregate multiscale context information required for classification and minimize the effect of noise via using the z-direction information. However, there are inception and residual modules in the coding path, which are some key differences from U-Net. In deep learning, deeper layers can lead to overfitting and vanishing gradient problems. The size of the receptive field is another important factor affecting network performance. The inception module allows information to pass through multiple sized cores in parallel, applying 1×1 convolution reduces the number of input feature maps before using large kernel convolution to effectively reduce the complexity of the model. Residual learning is able to alleviate the gradient vanishing problem. We apply skip connections similar to those in FCN,^29^ but add pyramid dilated deconvolution to further enhance the ability to represent multi-scale information in the spatial recovery path.

Because neurites only account for a small part of the data block, the categories of foreground (i.e., segmented neurites) and background are usually unbalanced, which may lead to prediction bias. The weighted cross-entropy (WCE) loss function^44^ and dice loss (DL) function^45^ reduce the impact of class level imbalance. We use a mixed loss function to combine these two loss functions to prevent class imbalance and maintain regional continuity. The WCE loss is

$$L_{\mathrm{WCE}}=\overset{L}{\underset{i}{\sum }}\overset{W}{\underset{j}{\sum }}\overset{H}{\underset{k}{\sum }}-\gamma \cdot g(x_{ijk})\log(p(x_{ijk})),\;\gamma =\frac{\sum_{i}^{L}\sum_{j}^{W}\sum_{k}^{H}g(x_{ijk})}{L\times W\times H}.$$ (3)

Then, the DL is

$$L_{\mathrm{DL}}=1-2\frac{\overset{L}{\underset{i}{\sum }}\overset{W}{\underset{j}{\sum }}\overset{H}{\underset{k}{\sum }}p(x_{ijk})g(x_{ijk})+\varepsilon }{\overset{L}{\underset{i}{\sum }}\overset{W}{\underset{j}{\sum }}\overset{H}{\underset{k}{\sum }}p(x_{ijk})+\overset{L}{\underset{i}{\sum }}\overset{W}{\underset{j}{\sum }}\overset{H}{\underset{k}{\sum }}g(x_{ijk})+\varepsilon },$$ (4)

where $p(x_{ijk})$ is the prediction probability of pixel x; $g(x_{ijk})$ is the corresponding pseudo-labels of foreground or background, and its values are 1 or 0 respectively; L is the length, W is the width, and H is the height of volume data; γ is the ratio of the number of voxels of foreground pseudo labels to the number of voxels of image volume data N; ε is the smoothing parameter set to 1. Thus, the combination loss function to train the model is $L=\sigma L_{\mathrm{WCE}}+L_{\mathrm{DL}}$, where σ is the weight of cross-entropy loss $L_{\mathrm{WCE}}$, which is used to balance the ratio of loss function.

### 3.3 Pseudo-Label Refinement

Some weak and uneven neuron signals are not detected in the pseudo-labels obtained by an adaptive enhancement filter, which reduce the network prediction accuracy of neuron images with low SNR. Therefore, it is necessary to improve the pseudo-labels for better segmentation. Huang et al.^46^ proposed a weak supervised learning method for neuron reconstruction. The region growing and skeleton method is used to mine more weak neurites from the probability map predicted by CNN iteratively. Here, we use the region growing method employed by Huang et al.^46^ to find more weak neurites from the probability map predicted by DDeep3M iteratively. The steps of the regional growth method are as follows:

1. Selection of seed region: According to the predicted probability map, the algorithm with maximum probability classification is used to generate the seed region of neurite. An adaptive threshold ρ is calculated for region growth in Step (2). ρ is the average value of voxels near the seed region (*Nreg*). *Nreg* is defined as follows:
  $$\mathrm{Nreg}=\{v\in N^{\prime }(v^{0})|v\notin \mathrm{Oreg},v^{0}\in \mathrm{Oreg}\},$$ (5)
  where $N^{\prime }(v^{0})$ is the 124 voxels neighborhood of $v^{0}$; *Oreg* is the seed region; and v, $v^{0}$ are the voxels.
2. Region growing: Based on neurite continuity, the seed region is expanded to include weaker neurites using region growing algorithm
  $$\mathrm{Greg}=\{v\in N(v^{0})|s(v)>\rho ,v^{0}\in \mathrm{Oreg}\}$$ (6)
  where $N(v^{0})$ is the 8 voxels neighborhood of $v^{0}$, s(v) is the probability map value of v; ρ is the adaptive threshold of region growth, and *Greg* represents the grown region.

Compared with the seed region, the grown region contained more voxels from neurites. Some weak neuron signals in the post-processing prediction graph are marked, which can be fused with the original image to obtain new enhanced training data for the next round of network training, to improve the accuracy of network prediction.

### 3.4 Image Enhancement

The output of DDeep3M network is the 3D probability map P(x), which is calculated by the SoftMax activation function after the 3D data block passes through convolution layers of the network. P(x)∈[0,1] denotes the probability that voxel x becomes the part of neuron. To use the prediction results, a natural method is to segment neurons directly from the prediction graph. However, because the accuracy of pseudo labels is not as good as manual annotation, especially in early iterations, some local details may be lost in the prediction graph. Therefore, we enhance the original image by fusing the prediction image and the original image to maintain the accurate neuron structure and suppress the noise signal effectively. By fusing the prediction result graph P(x) with the original image I(x) signal, the enhanced image block F(x) can be obtained, to preserve the local signal and the global structure at the same time (the image enhancement formula is shown below). When the enhanced image block F(x) is delivered to the Hessian module, a more complete neural community can be segmented and a better pseudo-label can be provided for the next iteration of network training. In the process of iterative learning, DDeep3M and Hessian modules complement and promote each other, thus gradually improving the performance of neuron segmentation.

Specifically, for an input image I(x), where x is a voxel, we identify the foreground voxels through the threshold δ (δ=2) selected by experience, to screen the probability map.^34^ If the foreground probability P(x) of voxel x is <δ, we set the intensity I(x) to zero, otherwise we keep the original intensity value unchanged. We use a step-edge function Θ(I(x)−δ) to describe an intermediate image. if P(x)>δ, Θ(I(x)−δ)=I(x); if P(x)≤δ, Θ(I(x)−δ)=0. Then, intermediate images and probability maps are fused, and the final image F(x) adjusted by our enhancement function is defined as

$$F(x)=\frac{\alpha }{\alpha +\beta }\cdot \Theta (I(x)-\delta )\cdot I(x)+\frac{\beta }{\alpha +\beta }\cdot \lfloor (1-\alpha )\cdot I_{M}\cdot P(x)\rfloor ,$$ (7)

where $I_{M}$ is the maximum intensity of the image, and α∈[0,1] is the weight controlling the contribution of voxel x in the original intensity, whereas β∈[0,1] is the weight controlling thecontribution of voxel x in the prediction graph, which were both set between 0 and 1. Using more reliable segmentation results as pseudo-labels, the segmentation network can be further trained to learn more distinctive and representative features for generating the probability map, which is conducive to the Hessian matrix based enhancement filter to segment more complete neurons in the next iteration. To explore the influence of parameter α for image enhancement, we adopt different values for α. In this paper, we empirically select α=0.8 to increase the robustness of the whole framework.

**Fig. 3:**
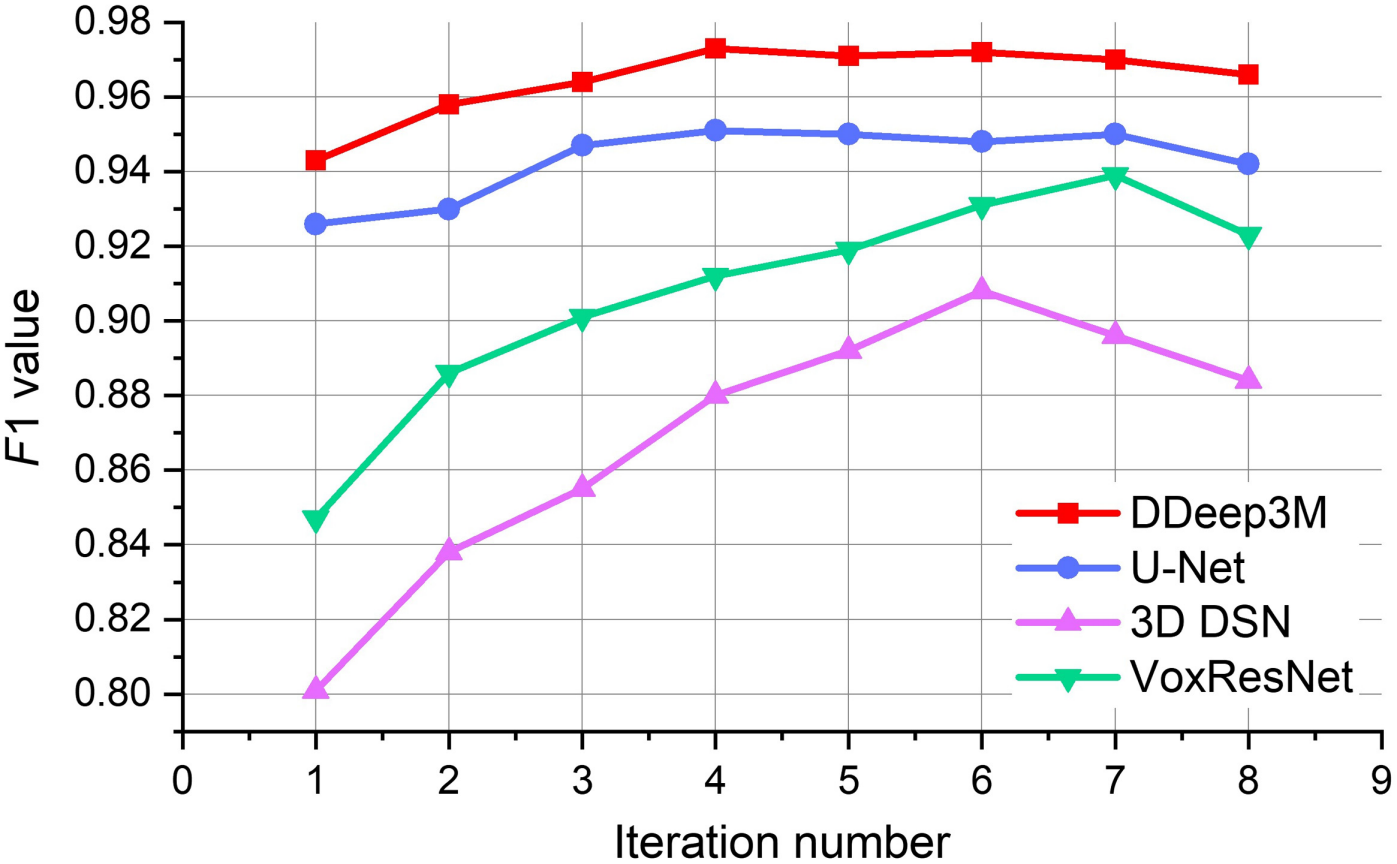
Comparisons of F1 value of the neuron segmentation at different iterations with four deep segmentation networks, respectively. After 4 iterations, DDeep3M network achieves the best segmentation result, with F1 value up to 0.973. Our DDeep3M+ effectively improves the neuron segmentation performance by combining any one of the three-neuron segmentation CNNs (U-Net,^30^ 3D DSN,^31^ and VoxResNet^32^).

During each iteration of the training process, we calculate the F1 value ($F_{t}$) of the test dataset. If the difference between $F_{t}$ and the F1 value from the previous iteration ($F_{t-1}$) is <0.005, and the difference between $F_{t}$ and the F1 value from the next iteration ($F_{t+1}$) is also <0.005 (i.e., $F_{t}-F_{t-1}<0.005$ and $F_{t+1}-F_{t}<0.005$), we consider the iteration to have converged to the optimal solution. Based on our experimental results (as shown in Fig. 3) and experience, we have found that the number of iterations required to converge to the best solution is usually between 4 and 6. Once convergence is achieved, the network training is considered complete.

## 4 Results

### 4.1 Datasets and Settings

We evaluate the proposed method and state-of-the-art segmentation methods based on deep learning on various 3D optical neuron datasets, including the fMOST datasets and the BigNeuron datasets. For the fMOST dataset, we select an image stack with the voxel size of 0.5  μm×0.5  μm×1  μm from the images of mouse neurons at the single fiber level for training and testing. The fMOST dataset used in the experiment is 3D volume data obtained from transgenic mouse Thy1 sample using two-photon fMOST (2P fMOST) system, which combines slicer and two-photon microscopy to realize continuous block imaging. In this study, the prediction set included the fMOST image stack of 3687×1224×1000  voxels from the V layer of the mouse cerebral cortex. During the training phase, we input a representative fMOST image stack of size 200×200×350  voxels into the DDeep3M framework for iterative network training. The image stack was divided into three datasets for training, validation, and testing, respectively. The training dataset comprised a volume of 200×200×200  voxels, whereas the validation and testing datasets were 200×200×50  voxels and 200×200×100  voxels in size, respectively. For each iteration, we fed the training and validation datasets, along with the corresponding pseudo-labels, separately into the neural network for training. The network’s performance on the test dataset and the ground truth data were used to evaluate its prediction accuracy at each iteration, which was then used to decide whether to continue training the network or terminate the iteration.

### 4.2 Training Details

The fMOST image stack is processed by a histogram equalization technology to strengthen the contrast, and then normalized by merely dividing the value of all pixels by 255. Finally, the data augmentation technique is applied to the original training set. Data augmentation can expand the effective size of training data, thereby decreasing the problem of over fitting. It is normally used to train CNN for image classification. By applying several spatial transformations to the input image, we enhance the training and test data. These transformations are a combination of horizontal and vertical mirrors, rotation +90  deg, −90  deg, and 180 deg in the XY plane, and flipping in the Z direction (horizontal mirror). A total of 5 datasets of 1000 images are employed in training. After all the converted data is provided to the network, we apply reverse conversion for each probability map. We take the predicted average of each transformation graph and the original graph as the final output of the boundary probability map.

We initialize the network by utilizing a Gaussian distribution with a standard deviation of 0.01. The mini batch size of our training model is 4, and the base learning rate is 0.01. The stochastic gradient descent optimization method with a momentum of 0.9 is adopted. Each model trains 30,000 iterations, and the mixed cross-entropy loss function is taken as our training objective. The network segmentation was trained and executed on a computer equipped with an Intel Xeon w-2123 CPU (64 GB RAM) and a Nvidia P5000 GPU (16 GB RAM). The prediction task for the fMOST image stack (3687×1224×1000  voxels) was completed by DDeep3M+ in ∼5  h, resulting in an average processing rate of 18.4 s per image.

### 4.3 Evaluation Metrics

To conduct quantitative comparisons among different segmentation methods, we first perform a binarization operation on the segmentation results obtained by different segmentation methods with a threshold value of 200, and then utilize common metrics to quantitatively evaluate the segmentation result between the ground truth labeled by human experts. They are precision, recall, F1, and Jaccard, and their definitions are given by

$$\text{Precision}=\frac{\mathrm{TP}}{\mathrm{TP}+\mathrm{FP}},$$ (8)

$$\text{Recall}=\frac{\mathrm{TP}}{\mathrm{TP}+\mathrm{FN}},$$ (9)

$$F1=2\cdot \frac{\text{Precision}\times \text{Recall}}{\text{Precision}+\text{Recall}},$$ (10)

$$\text{Jaccard}=\frac{\mathrm{TP}}{\mathrm{FP}+\mathrm{TP}+\mathrm{FN}},$$ (11)

here TP, TN, FP, and FN denote the true positive, true negative, false positive, and false positive.

To demonstrate the segmentation performance of our weakly supervised learning strategy, four widely-used deep segmentation networks, including U-Net, 3D DSN, VoxResNet, and DDeep3M, are tested to generate the neuron probability map in our framework. Eight iterations are tested on the fMOST dataset, and the F1 value improvement of segmentation results is shown in Fig. 3. It can be seen that our DDeep3M+ algorithm achieves the best segmentation effect, with the F1 value up to 0.973 after fourth iterations. After the fifth iteration, the F1 value decreases, and the network segmentation effect does not improve when the iteration number increases. It indicates that the prediction performance of the weakly supervised learning framework has reached the best after the fourth iteration. In addition, the performance of the DDeep3M network is better than the other three deep networks.

**Fig. 4:**
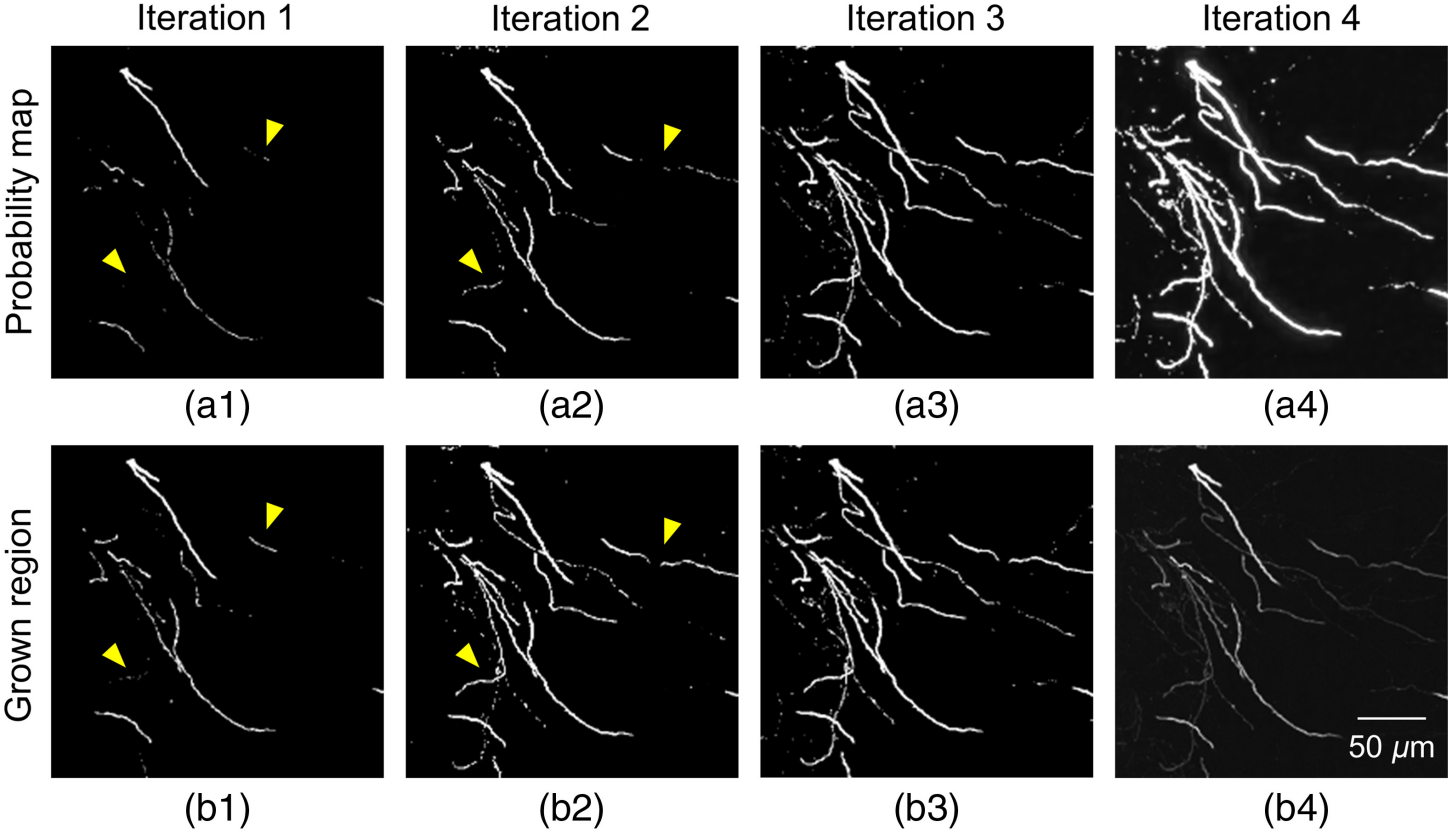
An example of pseudo-label refinement for weak neurite mining. (a1)–(a4) Probability map by DDeep3M at different iterations; (b1)–(b3) region grown to include nearby weak neurites; (c) original image. Yellow arrowheads point to the grown region. We iteratively mine more undetected weak neurites from the CNN-predicted probability map.

Moreover, the neuron segmentation results on a test block at different iterations are shown in Fig. 4, which depict the mining process of pseudo-label refinement. In Fig. 4, we can see that the prediction effect of DDeep3M network is more and more accurate, and many weak nerve fiber signals can be accurately segmented in the later stage, after several iterations of training.

### 4.4 Comparison with Other CNN Models

**Fig. 5:**
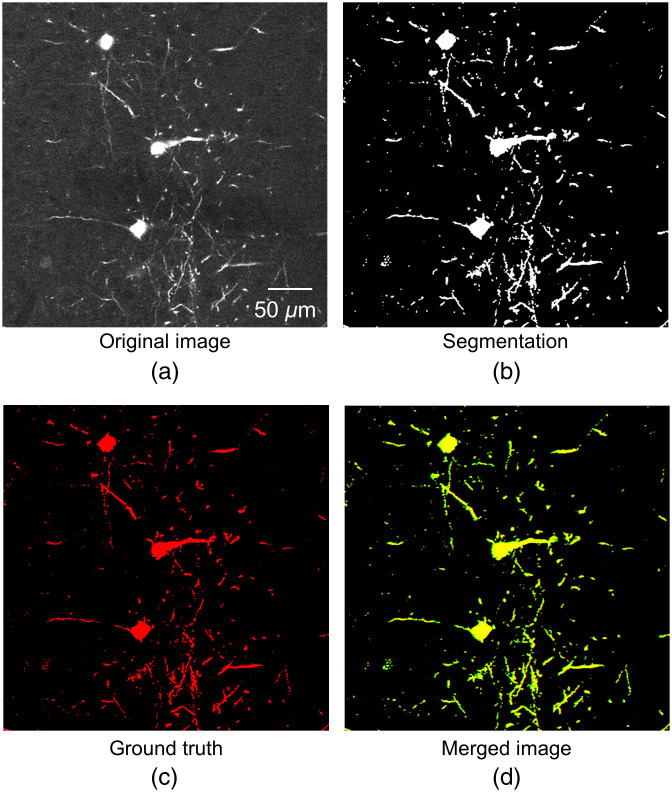
Validation of DDeep3M with the fMOST^16^ dataset after four iterations. (a) Original image, (b) segmentation of DDeep3M+, (c) ground truth, (d) merge of the segmentation (green) and the ground truth (red). The segmentation of neuron signal is realized accurately by DDeep3M+.

**Fig. 6:**
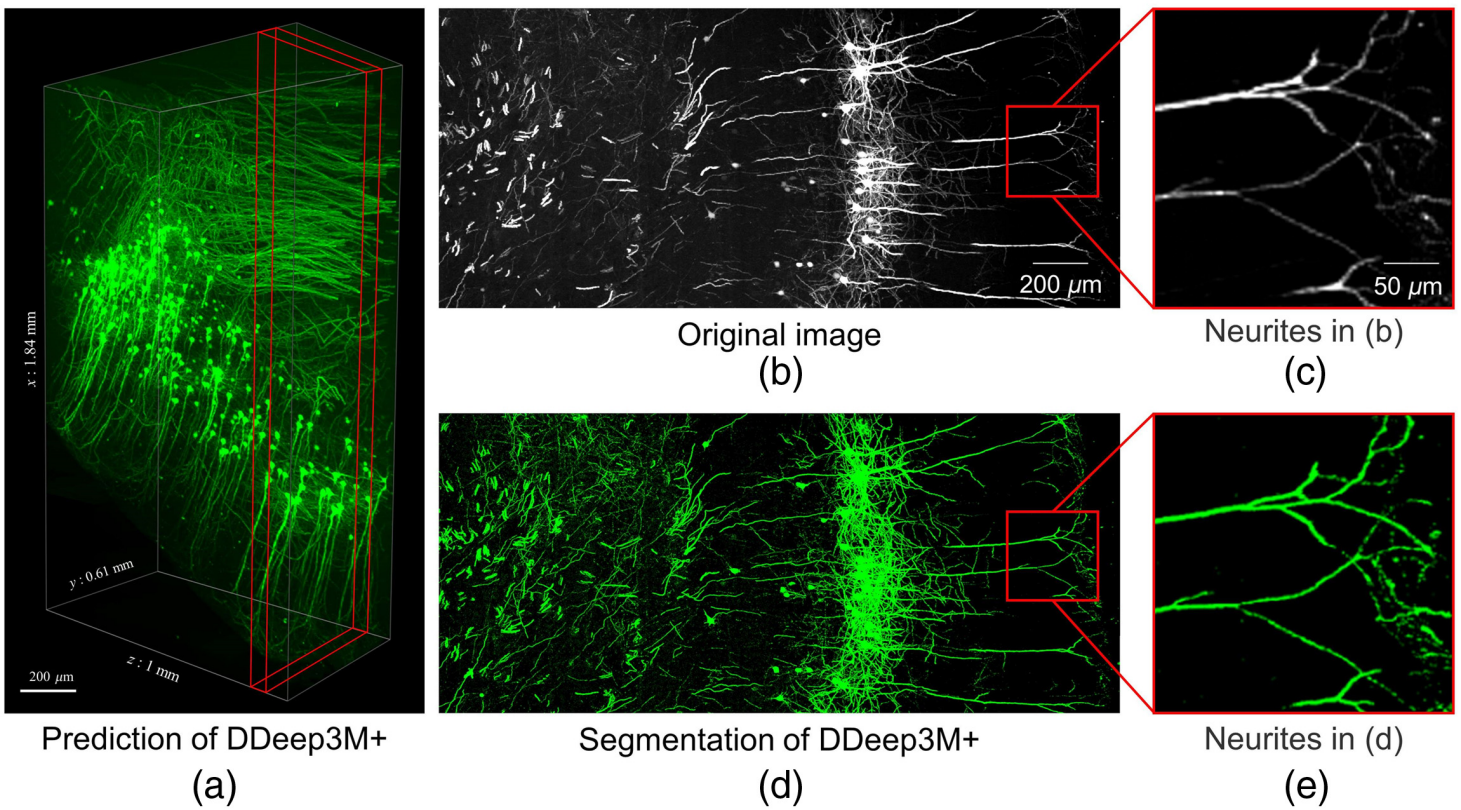
Prediction by DDeep3M+ on fMOST^16^ image stack. (a) 3D rendering of the prediction in a larger image stack. (b) Part of raw images in (a). (c) A magnified view of some of the nerve fibers in (b). The signal at the ends of the fibers is very weak and the connections are intermittent. (d) Segmentation of (b) with DDeep3M. (e) A magnified view of some of the nerve fibers in (d). Weak nerve fiber signals can be accurately identified and segmented, and the connections of nerve signals are continuous.

By comparing the detection performance of the proposed method against that of the common deep learning methods, we tested the effectiveness and accuracy of the proposed weakly supervised learning method for neuron detection and segmentation. The deep learning method used in this experiment is based on the supervised learning of the DDeep3M network. Figure 5 showed the segmentations of the proposed method on the fMOST datasets. Figure 6 displays the 3D rendering of the prediction results for a larger fMOST image stack (3687×1224×1000  voxels), as well as the maximum intensity projection of the original images and segmentations for 50 slices of the image stack. The proposed method detects almost all neurites from OM images. According to Table 1, the F1 score of the proposed method and the supervised deep learning method are 0.973 and 0.980, respectively; the recall and precision scores of the proposed method are 0.952 and 0.994, respectively; and the recall and precision scores of the deep learning method are 0.970 and 0.985, respectively. In Table 1, we also implement segmentation models that contain different parts of our DDeep3M+ modules in the fMOST image trainings, and the results show the performance for the different key structures. Table 1 shows that our DDeep3M+, which contains our enhancement filter to obtain pseudo-labels, has the best performance for fMOST neuron segmentation. We implement four segmentation networks with a threshold method (named Otsu) for pseudo-labels. And the results show that their performances are far worse than our DDeep3M+ method.

**Table 1:** Quantitative comparison results of neuron segmentation by different methods on fMOST^16^ data.

Methods		Precision	Recall	F1	Jaccard
DDeep3M+	Our	**0.994**	**0.952**	**0.973**	**0.947**
	Otsu	0.764	0.735	0.750	0.599
U-Net^30^	Our	0.993	0.913	0.951	0.907
	Otsu	0.599	0.836	0.598	0.536
3D DSN^31^	Our	0.994	0.836	0.908	0.831
	Otsu	0.386	0.926	0.545	0.374
VoxResNet^32^	Our	0.992	0.892	0.939	0.885
	Otsu	0.948	0.518	0.670	0.504
Supervised learning^42^		0.985	0.970	0.980	0.990

**Fig. 7:**
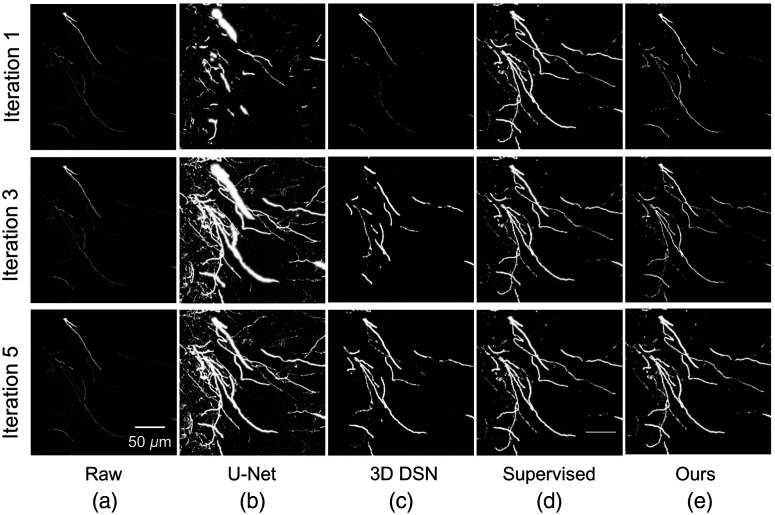
Segmentation performance of different networks: (a) raw images, (b) U-Net, (c) 3D DSN, (d) supervised method, and (e) our method. Neither U-Net^30^ network nor other weakly supervised learning method has an ideal segmentation effect on fMOST^16^ dataset. Our method segments neurons more completely and accurately compared to other methods. The proposed method is comparable to that of supervised deep learning method.

**Fig. 8:**
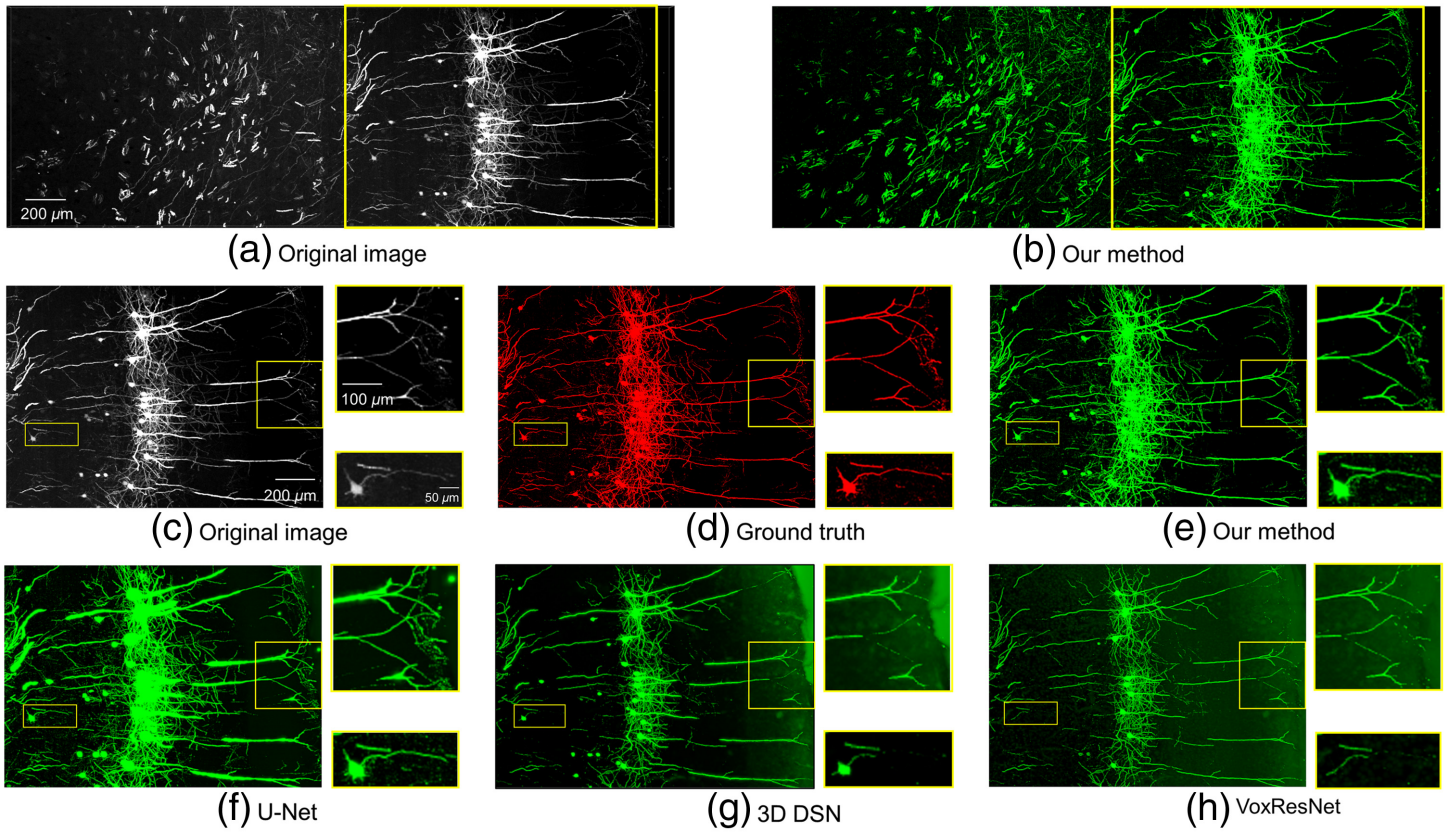
Enhancement comparison results in fMOST^16^ dataset. Images were acquired with a voxel size of 0.5  μm×0.5  μm×1  μm. (a) Original images and (b) enhanced results of our method. (c)–(h) Details of original images, ground truth images, enhanced results of our method, U-Net^30^ method, 3D DSN^31^ method, and VoxResNet^32^ method. Yellow box images show the situation of weak neurites.

Zhao et al.^47^ proposed a progressive learning method to reconstruct neurons via 3D DSN network from ultra-large scale optical microscopic images, and it is currently the excellent method for weakly supervised learning in the field of neuron segmentation. Figure 7 shows that neither U-Net segmentation network nor progressive learning method has an ideal segmentation effect on MOST dataset by comparing the segmentation performance of different network. We tested the proposed method and compared the final result with other three networks with a mouse brain dataset as shown in Fig. 8, which shows more details for comparison results between our method and other three segmentation networks. The result of proposed method shows more details of the fibers. As pointed out by the yellow box images in Fig. 8, the VoxResNet network was unable to detect neurites with weak or sudden changes in intensity. Similarly, it is difficult to trace some weak and uneven neurites from high noise images by 3D DSN network. In Fig. 8(f), there are some false positives in the result of U-Net network, which failed to trace some weak neurites precisely from the noisy background. Our method, employing weakly supervised learning, can accurately segment neuron cell body and fiber, and even some weak neurites can be accurately identified, and its segmentation performance is comparable to that of traditional deep learning method with manual annotations.^42^

### 4.5 Comparison on Other Datasets

**Fig. 9:**
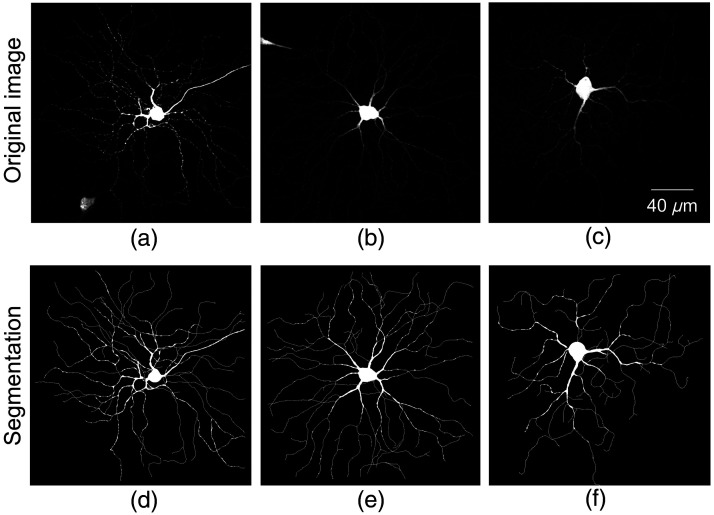
Segmentation by DDeep3M+ on BigNeuron image stacks. (a)–(c) Original images; (d)–(f) segmentations of (a)–(c). The proposed method also performs well on BigNeuron dataset.

We also test the performance of the proposed method on other datasets, as shown in Table 2. The proposed method also performs well on the BigNeuron datasets, with the highest F1 value up to 0.880, much higher than the 0.809 of U-Net network. The F1 value of U-Net, 3D DSN, and VoxResNet were 0.809, 0.816, and 0.649, respectively, whereas the performance of DDeep3M in our experiment is 0.855 (recall), 0.907 (precision), and 0.880 (F1 value). After the comparison of various quantitative metrics, the proposed method is better than other segmentation methods in all aspects. Figure 9 shows the segmented neurons on three test images from the BigNeuron dataset.

**Table 2:** Quantitative comparison results of neuron segmentation by different methods on BigNeuron^22^ data.

Methods	Precision	Recall	F1	Jaccard
DDeep3M+	**0.977**	**0.885**	**0.929**	**0.867**
U-Net^30^	0.921	0.722	0.809	0.680
3D DSN^31^	0.772	0.881	0.806	0.677
VoxResNet^32^	0.914	0.692	0.784	0.649
Supervised learning^42^	0.890	0.802	0.905	0.876

### 4.6 Enhancement Parameter

**Fig. 10:**
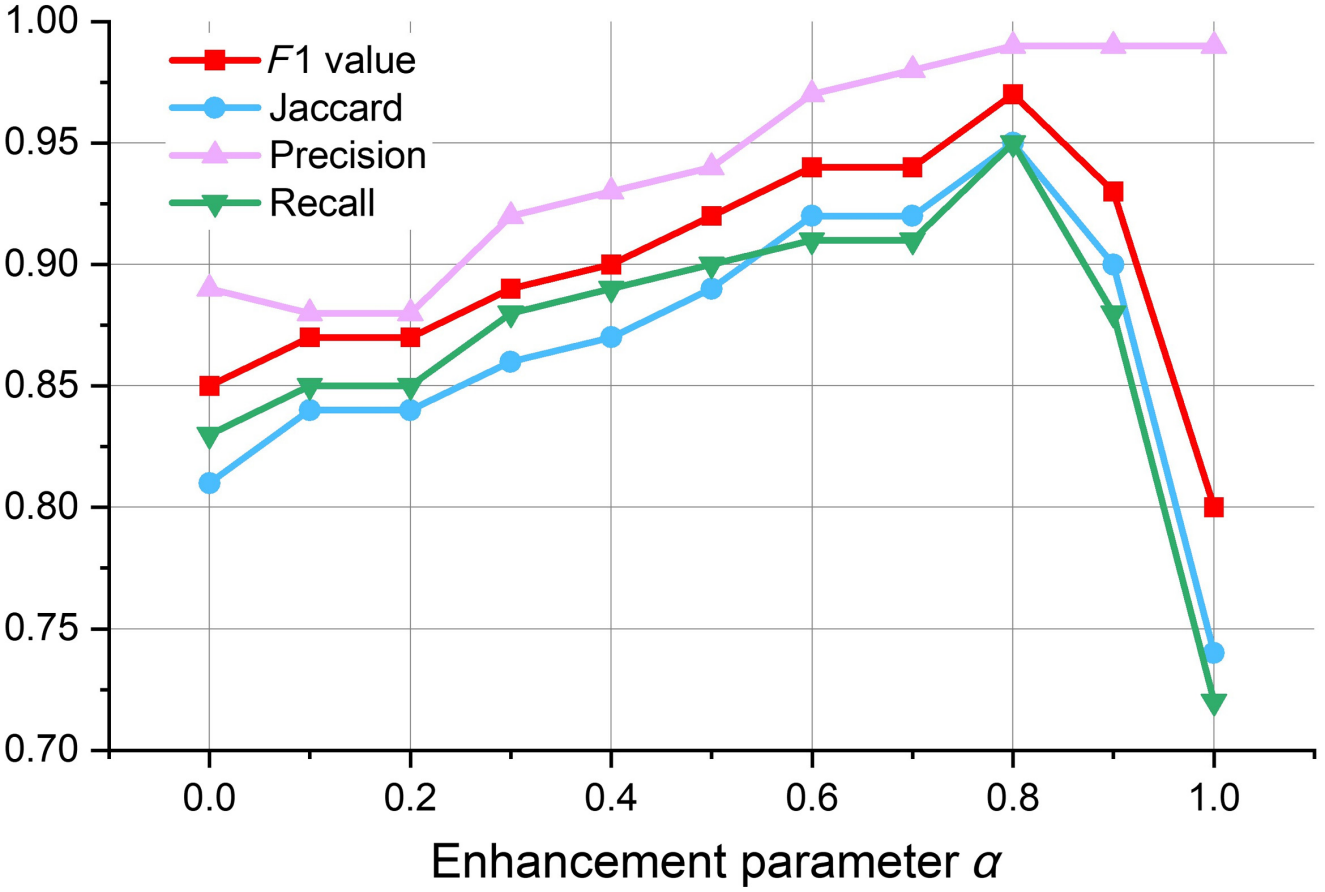
Neuron segmentation performance with different α in Eq. (7) for image enhancement. We choose the best model α=0.8 to reduce the impact of false positive prediction on probability map and improve the robustness of the whole framework.

To explore the influence of parameter α in enhancement function on image enhancement of our segmentation framework, we use different values of α and show the corresponding segmentation results of our method in Fig. 10. α=0 means that the original data block is directly used as the input of neuron segmentation. α=1 means that only the probability map is used as the input of neuron segmentation. This shows that the performance is improved by combining the probability map with the original image signals. The main reason is that the probability map reflects the long-range trajectory structure, whereas the original image signal carries more details of the neurites. The experimental results show that the segmentation performance can be improved by combining the probability map with the original image signal, which is mainly because the probability map reflects the long-range trajectory structures, whereas the original image signal has more details of subtle neural processes. Since we used pseudo labels to train the CNN model, its performance was limited when comparing it against the supervised learning method. To reduce the influence of false positive prediction on the probability map and combine with experimental verification, we chose α=0.8 in this paper to improve the robustness of the whole framework.

## 5 Discussion

Deep learning-based algorithms provide an effective and automatic method for neurite detection in highly noisy backgrounds. However, an accurate and robust estimation relies on large numbers of voxel-wise annotations. In recent years, the method of weakly supervised learning has gradually become a new trend in biomedical image segmentation. Huang et al.,^46^ Zhao et al.,^47^ and Chen et al.^48^ proposed three weakly supervised methods for neuron tracing and reconstruction. They solved the problem of the scarcity of manually annotated training samples and obtained better neurite detection results. Here, we employ a well-established CNN (DDeep3M) with inception and residual modules to achieve accurate, automatic, and universal neuron segmentation of 3D optical images for different types of neuron datasets with low and non-uniform signal strength. The proposed method is superior to several novel weakly supervised segmentation methods.

First, we apply the Hessian-based adaptive enhancement filter for 3D neuron images based on DT to obtain more accurate pseud-label. Our enhancement filter can preserve the neuron signals of subtle intensity. The enhanced images have shown better visual effect, higher image quality, more abundant information. In addition, the proposed algorithm has shorter time consumption. Thus, this algorithm will benefit the morphological study of neurons. The adaptive enhancement filter based on Hessian matrix is used to extract the global feature of the neuron voxel, which can effectively provide the approximate location of the neuron. However, some weak, disconnected, and heterogeneous neurites are not detected in the pseudo-labels obtained through the Hessian matrix, which reduces the accuracy of image segmentation for low SNR neuron images. Therefore, finer neuronal signals must be mined for better segmentation. Deep CNN has achieved impressive performance in both natural and medical image segmentation, and its efficient performance in extracting image features gives us the possibility of our work. Further use of the DDeep3M network on the adaptively enhanced image can detect almost all the neurites in the neuron image, including the difficult to identify thin neurites with relatively low intensity.

The initial training labels are constructed by the traditional image enhancement algorithm based on the neurite structure features. Then, the DDeep3M network improves prediction by iteratively optimizing the training labels and retraining model, while using regional growth to mine undetected weak neurites from the probability map predicted by DDeep3M. With more iterations of network training and image enhancement, the pseudo-labels are progressively refined and the DDeep3M model can gradually learn discriminative features of the neurites and background. Therefore, DDeep3M based segmentation method and traditional image enhancement technique can complement and promote each other. Finally, completer and more accurate neuron segmentation is obtained by our DDeep3M+ method.

In the training phase, deep learning completes the model establishment and parameter optimization, and in the inference phase, it completes the specific implementation and application. After carefully adjusting the weights, the neural network is basically a bulky and huge database. To make full use of the training results and complete the segmentation task in practical application, we used data quantification, layer fusion, and model pruning to optimize the inference process of the network. After the acceleration technology in inference phase, the speed of network prediction can be increased by about 12 times. At the same time, the F1 value of the prediction results did not decrease significantly, indicating that the inference speed was improved and the accuracy was not lost.

There are still some limitations of our work. (1) Our method is currently not as good as supervised learning methods in accuracy, although we have an advantage in the cost of manual labeling. In the future work, we need to make better use of the prior knowledge of neuronal signals and improve the architecture of deep networks to improve the prediction accuracy of weakly supervised learning and achieve more accurate segmentation of neuronal protrusions. (2) The selection of the value of the parameter in the enhancement formula in the work of image fusion is empirical, and there is no theoretical support. We choose α=0.8 as the weighting value of the original image according to the experience of the experiment. The enhancement result is the best, which is only obtained from limited discrete data. (3) The selection of the number of iterations in the whole weakly supervised learning scheme is also obtained according to the experience of experiments, which also lacks the explanation of the algorithm principle. (4) We have not carried out neuron reconstruction experiments on the results of neural network segmentation. In the future work, we will use neuronal images enhanced by neural networks for neuron tracing and reconstruction, so as to analyze the advantages of our method in promoting neuron tracing, reconstruction and visualization compared with other deep learning methods.

Compared to supervised learning methods, our method adapts to different types of optical neuron datasets without manual labeling, model redesign, or parameter tuning. The high accuracy results on challenging 3D optical images from different types of datasets demonstrate the accuracy and generalization of the proposed neurite segmentation method. The proposed weakly supervised learning framework can be extended to multi-modal biomedical big data, such as soma, vessels, and tumors, which helps life science experts to efficiently use deep learning techniques and promote further brain research. In addition, the source code of our method is open on the GitHub, which helps neuroscience researchers to easily use deep learning tools for their work on neuron segmentation and reconstruction.

## 6 Conclusions

We propose a weakly supervised deep learning method (i.e., DDeep3M+) for automatic neuron segmentation in 3D OM images. Our DDeep3M+ framework consists of an adaptive enhancement filter that extracts initial neuron signals as initial neurite segmentations and a deep segmentation network for precise neurite segmentations from low SNR optical images without manual labeling. The accuracy of segmentation is close to that one of the best supervised learning methods. The comparison between the proposed method and several new segmentation methods proves the superiority of the proposed method in the segmentation of neurons from low SNR images. The proposed framework is effective for both public fMOST and BigNeuron datasets, and can be used for automatic segmentation of super-large neuronal population in the entire brain region. The codes and the pretrained model weights of our method are available at GitHub, which will help neuroscience researchers to easily use deep learning tools for their work on neuron segmentation and reconstruction. We believe it will promote further brain studies, including neuron tracing, neuron reconstruction, and so on.

## Code, Data, and Materials Availability

The codes, datasets, and the pretrained model weights of our method are available at https://github.com/xiaoronghust/DDeep3Mplus/.
